# Physiological adaptations to 6 weeks of high‐intensity interval and moderate‐intensity continuous training in horses: A randomized crossover study

**DOI:** 10.14814/phy2.70785

**Published:** 2026-02-17

**Authors:** Kazutaka Mukai, Yuji Takahashi, Yusaku Ebisuda, Fumi Sugiyama, Toshinobu Yoshida, Hirofumi Miyata

**Affiliations:** ^1^ Sports Science Division Equine Research Institute, Japan Racing Association Shimotsuke‐shi Tochigi Japan; ^2^ Biological Sciences, Graduate School of Sciences and Technology for Innovation Yamaguchi University Yamaguchi‐shi Yamaguchi Japan

**Keywords:** cardiovascular adaptation, exercise performance, high‐intensity interval training, lactate metabolism, Thoroughbred

## Abstract

This study tested the hypothesis that 6 weeks of high‐intensity interval training (HIIT) would induce greater physiological adaptations than moderate‐intensity continuous training (MICT) in Thoroughbred horses. Seven untrained horses completed two distance‐matched treadmill training protocols (three sessions per week) in a randomized crossover design, separated by a three‐month washout: MICT (6 min at 70% $\dot{V}O_{2\max}$) and HIIT (6 × [30 s at 100% $\dot{V}O_{2\max}$ with 30 s at 30% $\dot{V}O_{2\max}$]). Incremental exercise tests were conducted at weeks 0, 3, and 6 to assess exercise performance and physiological responses. Mixed‐effects models were used to analyze the effects of time, protocol, and their interaction (*p* < 0.05). After 6 weeks, HIIT elicited greater improvements than MICT in run distance to exhaustion (+29% vs. +4.5%), speed at $\dot{V}O_{2\max}$ (+9.8% vs. +2.4%), and speed at maximal heart rate (+15% vs. +4.3%). Speed at 10 mmol/L of plasma lactate increased only after HIIT (+13% vs. +6.0%). Both protocols similarly improved $\dot{V}O_{2\max}$ (+13%) and maximal cardiac output (+7%–8%). In conclusion, despite being matched for total running distance, HIIT induced superior improvements in performance, cardiovascular function, and lactate kinetics compared with MICT, highlighting training intensity as a key determinant of training adaptations in horses.

## 1 INTRODUCTION

High‐intensity interval training (HIIT) has emerged as a highly effective training strategy in human athletes and is defined as repeated bouts of near‐maximal exercise (>80% of maximal oxygen consumption [$\dot{V}O_{2\max}$] or heart rate), interspersed with recovery periods (MacInnis & Gibala, 2017). When total work or energy expenditure is matched, HIIT induces physiological adaptations–including improvements in exercise performance, cardiorespiratory function, and aerobic metabolism–that are comparable to or greater than those achieved with moderate‐intensity continuous training (MICT), while requiring less total training time in humans (Daussin et al., 2008; MacInnis & Gibala, 2017; Ramos et al., 2015; Tabata et al., 1996). For example, Daussin et al. (2008) reported that 8 weeks of work‐matched HIIT protocols enhanced maximal cardiac output (Q_max_), $\dot{V}O_{2}$ kinetics, and skeletal muscle mitochondrial respiration, whereas MICT did not. Protocols eliciting $\dot{V}O_{2\max}$ or near‐maximal efforts are thought to strongly stimulate oxygen transport and both aerobic and glycolytic pathways, thereby inducing robust adaptations that enhance exercise performance (Buchheit & Laursen, 2013a).

Thoroughbred horses possess exceptional exercise performance, aerobic capacity, and skeletal muscle mass. In a 1200‐m race, their peak heart rate exceeds 210 bpm and blood lactate concentration surpasses 20 mmol/L, respectively (Mukai et al., 2007). To succeed in racing, horses must train at intensities close to race pace to adapt to the substantial mechanical and metabolic demands. High‐intensity exercise is also critical for activating fast‐twitch muscle fibers, which comprise more than 80% of the middle gluteal muscle in Thoroughbreds (Kawai et al., 2009). Previous studies in Thoroughbred horses have shown that high‐intensity training enhances whole‐body exercise performance and aerobic capacity (Kitaoka et al., 2012; Mukai, Ohmura, et al., 2021) and improves oxidative metabolism, glycolytic pathways, and lactate transporter expression in skeletal muscle (Eto et al., 2004; Kitaoka et al., 2011). These findings suggest that HIIT may be an efficient strategy for training racehorses. Indeed, we recently compared the acute responses of moderate‐intensity continuous exercise (MICE; 6 min at 70% $\dot{V}O_{2\max}$) and high‐intensity interval exercise (HIIE; 6 × 30 s at 100% $\dot{V}O_{2\max}$ with 30 s recovery at 30% $\dot{V}O_{2\max}$) and found that HIIE induced greater arterial hypoxemia, lactic acidosis and muscle glycogen depletion than MICE, while activating the AMPK signaling cascade and promoting mitochondrial biogenesis (Mukai et al., 2023).

Despite these promising acute responses, no study has systematically investigated whether HIIT produces superior long‐term training adaptations compared with MICT in Thoroughbred horses. This knowledge gap is important because optimizing training protocols for racehorses has direct implications for performance, safety, and the efficiency of training programs.

Therefore, the aim of this study was to compare physiological adaptations in Thoroughbred horses following 6 weeks of MICT and HIIT, matched for total running distance, in a randomized crossover design. We hypothesized that HIIT would induce greater improvements in exercise performance, cardiovascular capacity, and lactate metabolism than MICT, providing novel evidence to guide high‐performance equine training.

## 2 MATERIALS AND METHODS

All experimental protocols were reviewed and approved by the Animal Welfare and Ethics Committee of the Japan Racing Association (JRA) Equine Research Institute (Permit number: 2020‐11 and 2020‐12). All incisions for catheter placements were performed under local anesthesia using lidocaine, and every effort was made to minimize animal discomfort.

### 2.1 Horses

Eight untrained, healthy Thoroughbreds (5 geldings, 3 mares; mean ± SE age, 4.5 ± 1.3 years; body weight, 531 ± 79 kg at the onset of the study) were enrolled. To facilitate arterial catheterization, the carotid artery was surgically relocated from the carotid sheath to a subcutaneous location under sevoflurane anesthesia. At least 2 months after surgery, horses were habituated to running on a treadmill (Sato I, Sato AB, Uppsala, Sweden) while wearing an open‐flow mask (Pascoe et al., 1999). Horses were kept in a 17 × 22 m paddock for approximately 4 h/day for at least 12 weeks before incremental exercise tests (IETs) commenced. All horses were fed 1 kg oats, 1 kg pelleted feed (Power up horse II, Nosan Corporation, Yokohama, Japan), and 3 kg timothy hay in the morning, and 1 kg oats, 2 kg pelleted feed, and 3 kg timothy hay in the afternoon. Water was available ad libitum. Lameness evaluation, physical examination, cardiac auscultation, and ECG were performed by experienced veterinarians prior to the study and before every IET. Horses showing lameness or clinical abnormalities were excluded. One gelding developed right forelimb lameness during HIIT period (2nd block) and was excluded; thus, the statistical analyses were performed on 7 horses.

### 2.2 Experimental design

In a randomized crossover design, horses completed 6 weeks of distance‐matched treadmill training on a 6% incline, performed 3 days/week. Training protocols consisted of (1) MICT: 6 min at 70% $\dot{V}O_{2\max}$ (6.3–8.3 m/s) and (2) HIIT: 6 × (30 s at 100% $\dot{V}O_{2\max}$ [9.2–12.1 m/s] + 30 s at 30% $\dot{V}O_{2\max}$ [2.8–3.6 m/s]). On the other 4 days/week, horses were pastured for approximately 4 h/day and walked for 1 h/day in a walker. A 12‐week washout period separated two training blocks, during which horses walked for 1 h/day in a walker and/or were pastured for approximately 4 h/day.

### 2.3 Incremental exercise test (IET)

IETs were performed at week 0, 3, and 6. Training speeds corresponding to 70% and 100% $\dot{V}O_{2\max}$ were adjusted based on the results of IETs at week 0 and 3. Post‐training IETs were conducted 48 h after the last exercise bout to minimize acute exercise effects and prevent detraining, as described previously (Mukai, Kitaoka, et al., 2021; Perry et al., 2010). The IET protocol (Mukai et al., 2017) consisted of a 3‐min warm‐up at 4 m/s, followed by 2‐min steps at 1.7, 4, 6, 8, 10, 12, and 13 m/s on a 6% incline, until horses could no longer maintain their treadmill position despite humane encouragement (defined as exhaustion).

### 2.4 Measurement of oxygen consumption during IETs

Horses wore an open‐flow mask attached to a blower. Airflow was measured via pneumotachograph (LF‐150B, Vise Medical, Chiba, Japan) and a differential pressure transducer (TF‐5, Vise Medical). Gas concentrations were measured with O_2_ and CO_2_ analyzers (O_2_: FC‐10, Sable Systems International, NV; CO_2_: MG‐360, Vise Medical), calibrated with mass flow meters (CR‐300, Kofloc, Kyoto, Japan) using the N_2_‐dilution/CO_2_‐addition mass‐balance technique (Fedak et al., 1981). Signals were recorded at 200 Hz using commercial hardware and software (DI‐720 and Windaq Pro+, DATAQ, Akron, OH), and VO_2_ was calculated from the final 30 s of each step.

### 2.5 Blood sampling after training sessions

Once weekly during each training period, venous blood samples were collected from the jugular vein immediately after exercise. Samples were placed in heparinized tubes, centrifuged (1740 × g, 10 min), and analyzed for plasma lactate concentration using a lactate analyzer (Biosen S‐Line, EKF‐diagnostic GmbH, Barleben, Germany).

### 2.6 Blood sampling and heart rate measurements at IETs

Before each IET, an 18‐gauge catheter (Surflow, Terumo, Tokyo, Japan) was placed in the left carotid artery, and an 8‐F introducer (MO95H‐8, Baxter International, Deerfield, IL) in the right jugular vein. A Swan‐Ganz catheter (SP5107U, Becton, Dickinson and Company, Franklin Lakes, NJ) was advanced into the pulmonary artery via the introducer, with placement confirmed by pressure waveforms using a pressure transducer (P23XL, Becton, Dickinson and Company, Franklin Lakes, NJ).

Arterial and mixed‐venous blood samples were collected into heparinized syringes for the final 30 s of each step and at 1, 3, 5, and 10 min post‐exhaustion. Samples were kept on ice and analyzed immediately (blood gas analyzer: ABL800 FLEX; hemoximeter: ABL80 FLEX‐CO‐OX; Radiometer, Copenhagen, Denmark). Plasma lactate concentration was subsequently measured as described above. The speed eliciting a plasma lactate concentration of 10 mmol/L (VLA10) was calculated from the exponential speed‐lactate relationship for each IET.

Pulmonary arterial temperature (T_PA_) was measured using a cardiac output computer (COM‐2, Baxter International, Deerfield, IL) to correct blood gas measurements. Heart rate was continuously recorded (S810, Polar, Kempele, Finland) and averaged over the final 30 s of each step. Electrode sites were wetted and positioned on the saddle blanket and elastic girth for optimal signal detection.

### 2.7 Statistical analysis

Data are expressed as mean ± standard deviation (SD). Physiological variables were analyzed using mixed‐effects models with time, training protocol, and their interaction (time × protocol) as fixed effects, and horse as a random effect. Tukey's post hoc tests were applied when main effects or interactions were significant. Statistical analyses were performed using GraphPad Prism 10.5.0 (GraphPad Software, LLC., La Jolla, CA, USA). Statistical significance was set at *p* < 0.05.

## 3 RESULTS

### 3.1 Plasma lactate concentrations after exercise sessions during the training period

**FIGURE 1:**
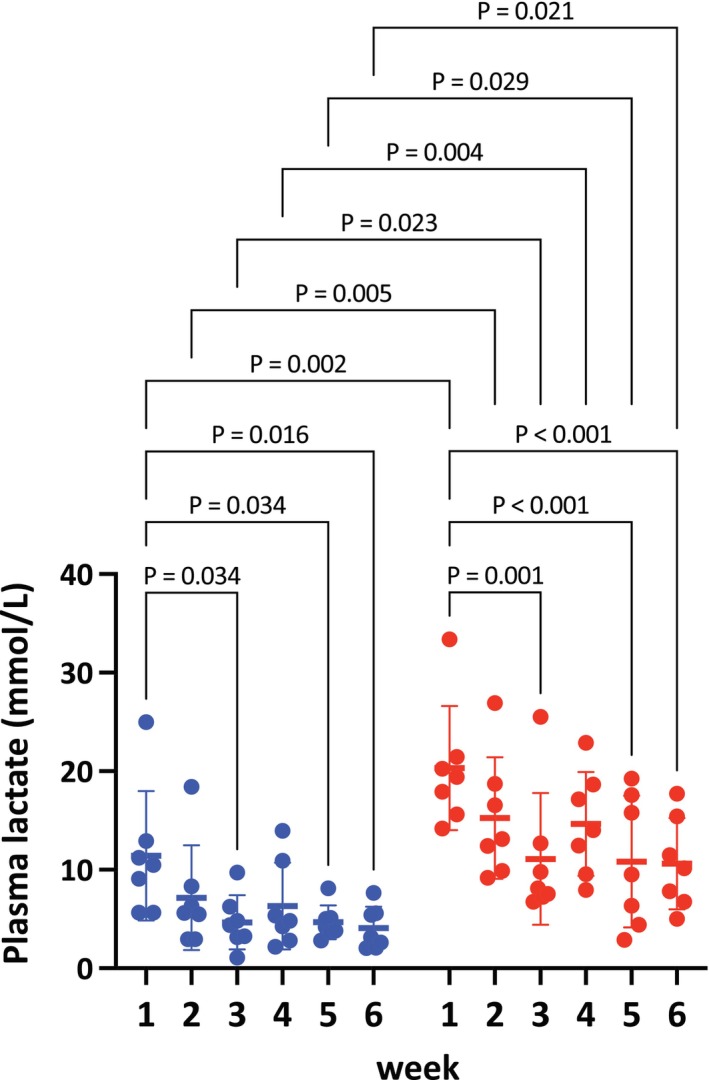
Plasma lactate concentrations during training sessions in MICT (blue) and HIIT (red) from week 1 to week 6. Each dot represents an individual horse. Data are presented as mean ± SD. Exact *p* values are shown above the brackets.

Significant main effects of time (*p* < 0.001) and protocol (*p* = 0.002) were observed. Throughout the training period, plasma lactate concentrations were constantly higher in the HIIT group compared to the MICT group as shown in Figure 1 (week 1, *p* = 0.001; week 2, *p* = 0.003; week 3, *p* = 0.017; week 4, *p* = 0.003; week 5, *p* = 0.022; week 6, *p* = 0.015).

At week 1, plasma lactate concentrations were 11.4 ± 6.6 mmol/L in the MICT group and 20.3 ± 6.3 mmol/L in the HIIT group. As training progressed, plasma lactate concentrations significantly decreased by week 3 to 4.7 ± 2.7 mmol/L in the MICT group (*p* = 0.034 vs. week 1) and 11.1 ± 6.7 mmol/L in the HIIT group (*p* = 0.001 vs. week 1).

At week 4, plasma lactate concentrations showed nonsignificant increases to 6.3 ± 4.4 mmol/L in the MICT group and 14.7 ± 5.3 mmol/L in the HIIT group. By week 6, plasma lactate concentrations gradually decreased again to 4.1 ± 2.2 mmol/L in the MICT group (*p* = 0.016 vs. week 1) and 10.6 ± 4.6 mmol/L in the HIIT group (*p* < 0.001 vs. week 1) (Figure 1).

### 3.2 Physiological variables at IETs following training

#### 3.2.1 Run distance

**FIGURE 2:**
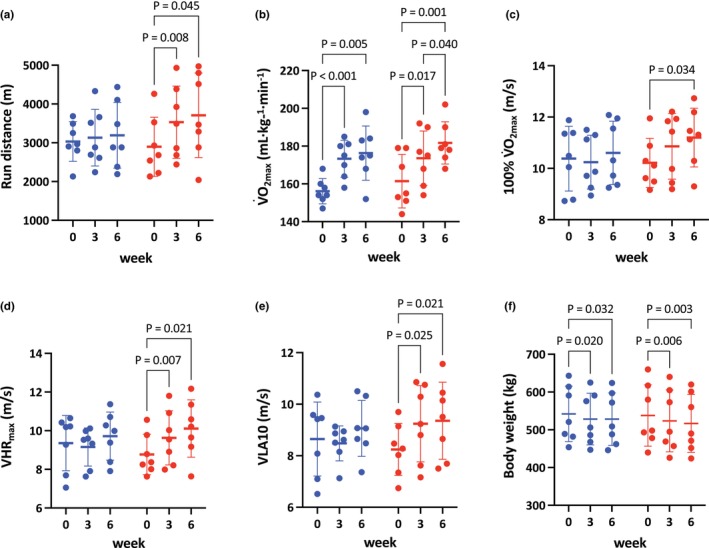
Run distance (a), maximal O_2_ consumption ($\dot{V}O_{2\max}$; b), speed eliciting $\dot{V}O_{2\max}$ (100% $\dot{V}O_{2\max}$; c), speed eliciting maximal heart rate (VHR_max_; d), speed eliciting 10 mmol/L of plasma lactate concentration (VLA10; e), and body weight (f) in MICT (blue) and HIIT (red) at week 0, 3, and 6. Each dot represents an individual horse. Data are presented as mean ± SD. Exact *p* values are shown above the brackets.

A significant interaction (*p* = 0.046) and a main effect of time (*p* = 0.005) were observed. Run distance significantly increased in the HIIT group at week 3 (+22%, *p* = 0.008) and week 6 (+29%, *p* = 0.045) compared to week 0. In contrast, no significant increases were observed in the MICT group (week 3, +2.8%, *p* = 0.706; week 6, +4.5%, *p* = 0.635), and no significant inter‐protocol differences were found at either week 3 (*p* = 0.393) or week 6 (*p* = 0.344; Figure 2a).

#### 3.2.2 $\dot{V}O_{2\max}$

There was a significant main effect of time (*p* < 0.001), with $\dot{V}O_{2\max}$ increasing similarly in both protocols at week 3 (MICT: +11%, *p* < 0.001; HIIT: +7.6%, *p* = 0.017) and week 6 (MICT: +13%, *p* = 0.005; HIIT: +13%, *p* = 0.001) (Figure 2b). No interaction was observed (*p* = 0.356), indicating changes in $\dot{V}O_{2\max}$ did not differ significantly between MICT and HIIT after training.

#### 3.2.3 Speed eliciting $\dot{V}O_{2\max}$ (100% $\dot{V}O_{2\max}$)

There was a significant main effect of time (*p* = 0.007), with 100% $\dot{V}O_{2\max}$ increasing only in the HIIT group at week 6 (+9.8%, *p* = 0.034), but not at week 3 (+6.3%, *p* = 0.173), nor in the MICT group at week 3 (−1.1%, *p* = 0.621) or week 6 (+2.4%, *p* = 0.627; Figure 2c). No significant interaction was observed (*p* = 0.051), indicating no significant difference between the two protocols following training (Figure 2c).

#### 3.2.4 Speed eliciting maximal heart rate (VHR_max_)

There was a significant interaction (*p* = 0.014) and a main effect of time (*p* < 0.001), with VHR_max_ increasing in the HIIT group at week 3 (+9.5%, *p* = 0.007) and week 6 (+15%, *p* = 0.021), but not in the MICT group at week 3 (−1.4%, *p* = 0.621) or week 6 (+4.3%, *p* = 0.252) (Figure 2d). However, no significant differences were observed between MICT and HIIT at week 3 (*p* = 0.481) or week 6 (*p* = 0.600).

#### 3.2.5 VLA10

There was a significant interaction (*p* = 0.036) and a main effect of time (*p* = 0.006), with VLA10 increasing in the HIIT group at week 3 (+12%, *p* = 0.025) and week 6 (+13%, *p* = 0.021), but not in the MICT group (week 3, −0.6%, *p* = 0.874; week 6, +6.0%, *p* = 0.503) (Figure 2e). No significant differences were observed between MICT and HIIT at week 3 (*p* = 0.248) or week 6 (*p* = 0.682).

#### 3.2.6 Body weight

There was a significant main effect of time (*p* < 0.001), with body weight decreasing in both groups at week 3 (MICT: −2.4%, *p* = 0.020; HIIT: −2.7%, *p* = 0.006) and week 6 (MICT: −1.6%, *p* = 0.032; HIIT: −3.8%, *p* = 0.003) compared to week 0 (Figure 2f). No significant interaction was observed (*p* = 0.227), indicating no significant difference between the MICT and HIIT groups following training.

#### 3.2.7 Q_max_

**FIGURE 3:**
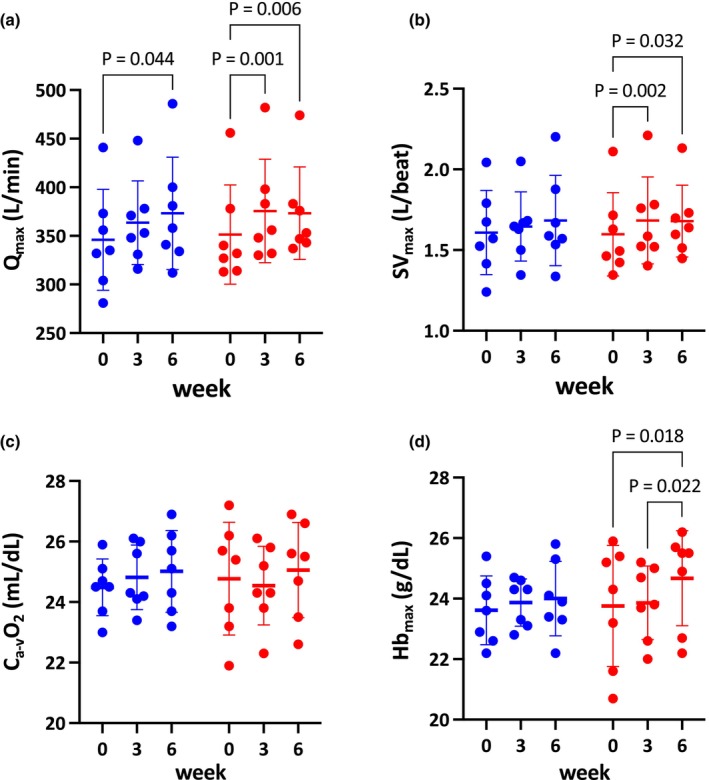
Maximal cardiac output (Q_max_; a), maximal stroke volume (SV_max_; b), arterial‐mixed venous O_2_ difference (C_a‐v_O_2_; c), and maximal hemoglobin concentration (Hb_max_; d) in MICT (blue) and HIIT (red) at week 0, 3, and 6. Each dot represents an individual horse. Data are presented as mean ± SD. Exact *p* values are shown above the brackets.

There was a significant main effect of time (*p* < 0.001), with Q_max_ increasing in the HIIT group at week 3 (+6.9%, *p* = 0.001) and week 6 (+6.5%, *p* = 0.006), and in the MICT group at week 6 (+7.9%, *p* = 0.044), but not at week 3 (+5.4%, *p* = 0.167) (Figure 3a). No significant interaction was observed (*p* = 0.502), indicating no significant difference between the MICT and HIIT groups following training.

#### 3.2.8 SV_max_

There was a significant main effect of time (*p* = 0.006), with SV_max_ increasing in the HIIT group at week 3 (+8.6%, *p* = 0.002) and week 6 (+8.2%, *p* = 0.032), but not in the MICT group (week 3, +3.7%, *p* = 0.684; week 6, +7.4%, *p* = 0.230) (Figure 3b). No significant interaction was observed (*p* = 0.535), indicating no significant difference between the MICT and HIIT groups following training.

#### 3.2.9 Arterial‐mixed venous O_2_ difference (C_a‐v_O_2_)

No significant interaction (*p* = 0.467) or main effect of time (*p* = 0.691) was observed, indicating that C_a‐v_O_2_ did not change in either group at week 3 (MICT: +1.5%; HIIT: −0.8%) or week 6 (MICT: +2.2%; HIIT: +1.4%) (Figure 3c).

#### 3.2.10 Maximal hemoglobin concentration ([Hb_max_])

A significant main effect of time was observed (*p* = 0.022), with [Hb_max_] significantly increasing only in the HIIT group at week 6 (+3.9%, *p* = 0.018), and not at week 3 in either group (MICT: +1.2%, *p* = 0.808; HIIT: +0.7%, *p* = 0.945), nor in the MICT group at week 6 (+1.6%, *p* = 0.245) (Figure 3d).

## 4 DISCUSSION

### 4.1 Exercise performance and cardiovascular adaptations

The main findings of this study were that 6 weeks of HIIT induces broader and more integrated physiological adaptations than MICT in Thoroughbred horses, when training distance is matched.

In human athletes, HIIT protocols that reach $\dot{V}O_{2\max}$ are considered necessary to maximally stimulate the oxygen transport system and induce robust improvements in cardiovascular capacity and exercise performance (Billat, 2001; Buchheit & Laursen, 2013a; Laursen, 2010). Our results extend these findings to equine athletes, showing that repeated near‐maximal efforts similarly enhance central circulatory function in horses. This interpretation is supported by our previous acute study using the same exercise protocols, in which HIIE achieved higher peak HR (214 vs. 196 bpm in MICE) and greater muscle glycogen depletion (65% vs. 79% of pre‐exercise level) than MICE (Mukai et al., 2023), suggesting stronger recruitment of fast‐twitch glycolytic fibers. Given that >80% of the middle gluteal muscle in Thoroughbreds consists of type II fibers (Kawai et al., 2009), the high‐intensity stimulus likely activated these fibers extensively and induced both metabolic and cardiovascular adaptations in this study.

Interestingly, both MICT and HIIT similarly improved $\dot{V}O_{2\max}$, Q_max_, whereas indices reflecting submaximal performance (100% $\dot{V}O_{2\max}$, VLA10, and VHR_max_) improved only after HIIT. This distinction suggests that while central adaptations can be induced by both MICT and HIIT, efficiency‐related adaptations such as running economy and lactate threshold are preferentially enhanced by HIIT. In humans, running economy has traditionally been linked to endurance training distance, but recent evidence indicates that HIIT can further augment this parameter through neuromuscular and metabolic pathways (Barnes & Kilding, 2015; McCann & Higginson, 2008). Although biomechanical adaptations may play a role, previous studies have reported inconsistent associations between running economy and biomechanical variables (Lake & Cavanagh, 1996). We did not directly assess biomechanical parameters in the present study, but the improvement in submaximal efficiency observed after HIIT suggests a role for metabolic remodeling, particularly within fast‐twitch fibers.

### 4.2 Determinants of VO_2max_

From the perspective of oxygen transport, $\dot{V}O_{2\max}$ is determined by Q (= SV × HR) and arterial‐mixed venous oxygen difference (C_a‐v_O_2_), according to the Fick equation. In humans, increases in $\dot{V}O_{2\max}$ following endurance training are primarily driven by increased stroke volume and red cell mass rather than peripheral oxygen extraction capacity (Gibala, 2021; Lundby et al., 2017). Our data support this view: both MICT and HIIT increased $\dot{V}O_{2\max}$ and Q_max_, with no significant change in C_a‐v_O_2_. This finding aligns with previous reports in humans (Gibala & MacInnis, 2022; Warburton et al., 2004), indicating that central oxygen delivery remains the principal limiting factor of $\dot{V}O_{2\max}$ in both species (Jones & Lindstedt, 1993). However, a previous study has reported that HIIT improved $\dot{V}O_{2\max}$ and SV_max_ in trained humans whereas MICT did not, suggesting that the training status of subjects influences the relative contribution of intensity (Helgerud et al., 2007). In our untrained horses, most of the cardiovascular improvements occurred in the first 3 weeks of training, with further increases in $\dot{V}O_{2\max}$ only in the HIIT group thereafter. These results suggest that the physiological ceiling may be reached earlier with MICT, whereas HIIT continues to provide sufficient stimulus even in partially adapted individuals. Furthermore, plasma lactate concentrations at week 5 and 6 were considerably lower than those at week 1 in both MICT and HIIT, which suggests that the metabolic stimulus in both protocols was gradually attenuated during the training period, despite resetting the training intensity after 3 weeks. Although lactate is not the sole determinant of training adaptation, it serves as a valuable marker for exercise intensity. We previously reported a positive correlation between plasma lactate concentration and post‐exercise PGC‐1α mRNA, a master regulator of mitochondrial biogenesis (Kitaoka et al., 2020). Similarly, Perry et al. (2010) observed that PGC‐1α mRNA response diminished as skeletal muscle adapted to repeated HIIT in humans. These findings may explain the reduced magnitude of physiological improvements in the latter half of training in our study.

### 4.3 Hematological adaptations

An additional finding was that [Hb_max_] increased after HIIT but not after MICT. Although hematological adaptations to HIIT remain underexplored, several weeks of endurance training are generally associated with red cell mass and hemoglobin concentration (Mairbaurl, 2013). Given the popularity of altitude/hypoxic training among elite athletes, hypoxia is recognized as a key driver of hematological adaptation (Wehrlin et al., 2006). In our previous study, only HIIE induced hypoxemia and increased post‐exercise hypoxia‐inducible factor (HIF)‐1α mRNA in skeletal muscle (Mukai et al., 2023). Taken together, repeated hypoxic stimuli during HIIE may have contributed to the increased [Hb_max_] after 6 weeks of HIIT. Importantly, hematological changes typically require >2–3 weeks to manifest, which is consistent with our results that [Hb_max_] did not increase after 3 weeks of HIIT.

### 4.4 Lactate metabolism

The improvement in VLA10 in the HIIT group suggests that enhanced lactate clearance and delayed accumulation during high‐intensity exercise. Lactate metabolism is influenced by the balance of glycolytic flux, oxidative capacity, and lactate transport across membranes via monocarboxylate transporters (MCTs). MCT1, expressed in oxidative fibers, facilitates lactate uptake, whereas MCT4, expressed in glycolytic fibers, promotes lactate efflux (Bonen, 2000; Bonen et al., 2000). In human, 6 weeks of HIIT increased citrate synthase activity, COX IV content, MCT1 and MCT4 expression, thereby reducing glycogenolysis and lactate accumulation (Perry et al., 2008). In horses, high‐intensity training for 18 weeks increased MCT1 and 4 expression and oxidative enzyme activity, with detraining for 6 weeks selectively reducing MCT4 but not MCT1 (Kitaoka et al., 2011). Together with our previous acute data showing that HIIE induced lactic acidosis, AMPK activation, and downstream mitochondrial biogenesis (Mukai et al., 2023), these findings support that a mechanistic model in which repeated HIIT enhances oxidative metabolism, increases lactate transporter capacity, and thereby improves whole‐body lactate kinetics.

### 4.5 Musculoskeletal injury risk

While there was no musculoskeletal injury in the MICT group, we experienced a suspensory ligament injury in the HIIT group. In human athletes, HIIT protocols can impose abrupt high mechanical loads and rapid accelerations, which increase musculoskeletal injury risk (Buchheit & Laursen, 2013b). Epidemiological studies in racehorses also report that insufficient and excessive exposure to high‐speed exercise increases the risk of catastrophic musculoskeletal injury (Hitchens et al., 2019; Verheyen et al., 2006). In our study, the use of untrained horses and the abrupt increase in training intensity may have contributed to this outcome. Furthermore, the repeated acceleration‐deceleration nature of HIIT may impose additional strain on musculoskeletal structures, though this has yet to be systematically investigated in horses. These observations emphasize the need for caution when implementing HIIT protocols in practice.

### 4.6 Limitations

The relatively small sample size (*n* = 7) may limit generalizability of the findings. A formal a priori power analysis was not performed because of practical and ethical constraints associated with the use of Thoroughbred horses, including availability, cost, and welfare considerations, which limited the achievable sample size. Similar sample sizes have been used in previous controlled physiological training studies in horses (Davis et al., 2023; Mukai, Kitaoka, et al., 2021). Regarding the randomized crossover design, we have clarified that this approach enhanced statistical power by allowing each horse to serve as its own control, thereby reducing inter‐individual variability in physiological responses and improving the sensitivity to detect training‐induced differences between protocols. Furthermore, we attempted to balance sex distribution (4 geldings and 3 mares) to minimize sex‐related differences; however, sex‐specific training adaptations cannot be entirely excluded. Nevertheless, previous studies suggest that sex differences in equine exercise performance are minimal compared with those observed in humans (Entin, 2007; McClelland & Weyand, 2022; Senefeld et al., 2021). Another limitation is the absence of direct mechanistic analyses of skeletal muscle (e.g., molecular and/or histological assays), which prevented confirmation of the mechanistic pathways inferred from our previous work. Finally, while our data support exercise intensity as the key driver of adaptation, the specific contributions of repeated acceleration and deceleration intrinsic to HIIT remain unsolved. Further research directly comparing high‐intensity continuous training with HIIT is required to clarify the underlying mechanism.

## 5 CONCLUSIONS

When matched for training distance, 6 weeks of HIIT produced superior improvements in exercise performance, aerobic capacity, lactate metabolism, and hematological parameters compared with MICT in Thoroughbred horses. These results highlight the importance of training intensity in driving integrated adaptations and suggest that HIIT represents a highly effective training strategy for Thoroughbred horses. Nevertheless, the potential risk for increased musculoskeletal injury should be carefully considered when implementing HIIT in racehorse training programs.

## AUTHOR CONTRIBUTIONS

K.M. and H.M. conceived and designed research; K.M., Y.T., Y.E., F.S., and T.Y. performed experiments; K.M. analyzed data; K.M. interpreted results of experiments; K.M. prepared figures; K.M. drafted manuscript; K.M. edited and revised manuscript; K.M., Y.T., Y.E., F. S., T.Y., and H.M. approved the final version of manuscript.

## FUNDING INFORMATION

This study was funded by the JRA.

## CONFLICT OF INTEREST STATEMENT

K.M., Y.T., Y.E., F.S., and T.Y. were the employees of the JRA. The JRA had no role in the design of the study; in the collection, analysis, or interpretation of data; or in the writing of the manuscript.

## ETHICS STATEMENT

All experimental protocols were reviewed and approved by the Animal Welfare and Ethics Committee of the JRA Equine Research Institute (Permit number: 2020‐11 and 2020‐12).

## Data Availability

Original data are available on request to the corresponding author.
